# Non-inflammatory tumor microenvironment of diffuse intrinsic pontine glioma

**DOI:** 10.1186/s40478-018-0553-x

**Published:** 2018-06-28

**Authors:** Grant L. Lin, Surya Nagaraja, Mariella G. Filbin, Mario L. Suvà, Hannes Vogel, Michelle Monje

**Affiliations:** 1 Department of Neurology, Stanford University, Stanford, CA 94305 USA; 2 Department of Pediatric Oncology, Dana-Farber/Boston Children’s Cancer and Blood Disorder Center and Harvard Medical School, Boston, MA USA; 3 Department of Pathology, Massachusetts General Hospital, Boston, MA USA; 4 Klarman Cell Observatory, Broad Institute of Harvard and Massachussetts Institute of Technology (MIT), Cambridge, MA 02142 USA; 5 Department of Pathology, Stanford University, Stanford, CA 94305 USA; 6 Department of Pediatrics, Stanford University, Stanford, CA 94305 USA

**Keywords:** Diffuse intrinsic pontine glioma, Tumor-associated macrophage, Tumor-infiltrating lymphocyte, Glioma, Glioblastoma, Immune microenvironment

## Abstract

Diffuse intrinsic pontine glioma (DIPG) is a universally fatal malignancy of the childhood central nervous system, with a median overall survival of 9–11 months. We have previously shown that primary DIPG tissue contains numerous tumor-associated macrophages, and substantial work has demonstrated a significant pathological role for adult glioma-associated macrophages. However, work over the past decade has highlighted many molecular and genomic differences between pediatric and adult high-grade gliomas. Thus, we directly compared inflammatory characteristics of DIPG and adult glioblastoma (GBM). We found that the leukocyte (CD45+) compartment in primary DIPG tissue samples is predominantly composed of CD11b + macrophages, with very few CD3+ T-lymphocytes. In contrast, T-lymphocytes are more abundant in adult GBM tissue samples. RNA sequencing of macrophages isolated from primary tumor samples revealed that DIPG- and adult GBM-associated macrophages both express gene programs related to ECM remodeling and angiogenesis, but DIPG-associated macrophages express substantially fewer inflammatory factors than their adult GBM counterparts. Examining the secretome of glioma cells, we found that patient-derived DIPG cell cultures secrete markedly fewer cytokines and chemokines than patient-derived adult GBM cultures. Concordantly, bulk and single-cell RNA sequencing data indicates low to absent expression of chemokines and cytokines in DIPG. Together, these observations suggest that the inflammatory milieu of the DIPG tumor microenvironment is fundamentally different than adult GBM. The low intrinsic inflammatory signature of DIPG cells may contribute to the lack of lymphocytes and non-inflammatory phenotype of DIPG-associated microglia/macrophages. Understanding the glioma subtype-specific inflammatory milieu may inform the design and application of immunotherapy-based treatments.

**Electronic supplementary material:**

The online version of this article (10.1186/s40478-018-0553-x) contains supplementary material, which is available to authorized users.

## Introduction

Diffuse intrinsic pontine glioma (DIPG) is a devastating tumor that arises in the ventral pons, chiefly during childhood, and is universally fatal. Currently, the standard of care is radiotherapy, which offers temporary stabilization or reduction of symptoms and extends median survival by approximately 3 months [21]. Overall median survival remains 9–11 months with less than 10% of children surviving beyond 2 years [7, 20]. Over the past decade, comprehensive efforts to characterize the genomic and epigenomic features of DIPG and other pediatric tumors have revolutionized our understanding of this disease [29]. Pediatric high-grade gliomas are genomically distinct tumors when compared to adult glioblastoma (GBM) [47]. A highly recurrent mutation in genes encoding histone-3 (H3K27M) is found in ~ 80% of all DIPG and also the majority of thalamic and spinal cord gliomas of childhood [2, 9, 11, 22, 44, 45, 48, 50, 53, 54]; these H3K27M mutant gliomas were recently reclassified as “diffuse midline gliomas, H3K27M mutant subtype” [23]. Broadly, analysis of immune infiltrates of pediatric central nervous system tumors suggests the immune compartments are less predictive of tumor grade or prognosis, compared to adult gliomas [37]. For example, pediatric GBM samples exhibit a muted immune functional phenotype compared to pediatric pilocytic astrocytomas and ependymomas [15], but the immune state of DIPG has not been similarly analyzed to date. DIPG exhibits a unique mutational profile and transcriptional state [27, 29], raising the question of a similarly unique immune microenvironment. As interest in the clinical application of immunotherapeutic approaches for DIPG grows, it is critically important to understand how potential differences in the DIPG immune microenvironment could influence treatment efficacy. For example, the presence or absence of lymphocytes would inform the consideration of strategies such as checkpoint inhibition.

Glioma-associated microglia/macrophages (GAMs) have received interest as a potential therapeutic target. In patients with adult GBM, expression of specific immune-associated gene sets are correlated with overall survival [52]. Moreover, adult GBM-associated macrophages have been implicated in supporting tumor cell proliferation, invasion, and survival as well as tumor angiogenesis [13, 17]. Targeting GAMs with CSF1R antagonism has demonstrated efficacy in a number of adult GBM preclinical models by affecting GAM activation state [39], an effect mediated by the tumor microenvironment [41]. CSF1R antagonism also potentiates the effect of radiotherapy in adult GBM preclinical models [46]. While evaluation of the clinical potential of CSF1R- and other GAM-targeting therapies is ongoing [3, 5], it is clear that GAMs are a critical component of the glioma microenvironment. Moreover, GAM activation state has been identified as an important factor in effective immunotherapy treatment [43], so understanding their phenotype across different tumors may help guide clinical translation of immune-targeting therapies.

In DIPG, little is known about the state of microglia and macrophages: due to the sensitive location and diffusely infiltrative nature of the tumor, resection is not possible, and the biopsy of DIPG has historically been uncommon [4, 23, 29, 38]. We have previously demonstrated that human DIPG samples demonstrate substantial immunoreactivity for the macrophage/microglial markers CD68 and CD163 [6], illustrating that GAMs are a large fractional component of the DIPG microenvironment. Here, we isolate GAMs from primary DIPG and adult GBM, as well as microglia from normal pediatric cortex samples for whole transcriptome analysis. We demonstrate that while DIPG-associated macrophages exhibit some gene expression programs similar to adult GBM-associated macrophages, they express substantially fewer inflammatory cytokines and chemokines compared to adult GAMs. Proteomic analyses reveal that patient-derived DIPG cultures produce markedly fewer cytokines and chemokines when compared to patient-derived adult GBM cultures, a finding corroborated by primary DIPG tissue bulk and single-cell RNA sequencing data. We also observed minimal lymphocytic infiltration in both primary DIPG autopsy and diagnostic biopsy samples. Together, these data suggest that DIPG and DIPG-associated macrophages are less inflammatory than adult GBM and adult GAMs.

## Materials and methods

### Acquisition and processing of human tissue samples

All human tissue studies were performed with informed consent and in accordance with Institutional Review Board (IRB)-approved protocols. Autopsy and biopsy tissue samples were processed as previously described [26], with some modifications. Briefly, tissue was minced finely, enzymatically dissociated in a collagenase/dispase + DNAse solution, triturated, and filtered through a 100μm filter to obtain a single cell solution. Debris was removed by centrifugation using a 0.9 M sucrose gradient, and red blood cells were removed using ACK lysis. Live cells were quantified via trypan blue exclusion, and resuspended for fluorescence-activated analysis and sorting.

### Fluorescence-activated cell sorting and analysis

Isolated single cells were resuspended in flow cytometry buffer (2% BSA, 10μM HEPES in HBSS without calcium/magnesium) at approximately 10^7^ cells/mL and processed at 4 °C. Cells were blocked with mouse IgG isotype control (ThermoFisher) and rat IgG isotype control (R&D Systems) as appropriate. Cells were then incubated with the appropriate conjugated primary antibodies (FITC-anti-CD45, BD Pharmigen 555482; PE-Cy7-anti-CD11b, BD Pharmigen 557743; PE-anti-CD3, Biolegend 300308; APC-Cy7-anti-CD31, Biolegend 303120) for 1 h. Next, cells were stained using the LIVE/DEAD Fixable Dead Cell Stain Kit (Invitrogen) according to the manufacturer’s instructions. Immediately before analysis and sorting, DAPI was added to the flow cytometry buffer. Cells were sorted and analyzed on a BD FACSAria II or FACSAria Fusion. Cells were identified and gated for size, singularity, cell viability, and all surface-marker expression was gated with appropriate full-minus-one controls. Cells were sorted into cold catching medium and a fraction was removed for cell purity analysis. Isolated cells were lysed in TRIzol (Invitrogen), and stored at − 80 °C until further processing.

### RNA sequencing

Total RNA was extracted by chloroform extraction into the RNA Clean & Concentrator Kit with DNAse (Zymo Research), and examined for quality using an Agilent Bioanalyzer 2100 platform. First and second-strand cDNA synthesis and SPIA amplification was performed with the Ovation RNAseq system V2 (Nugen) following the manufacturer’s instructions, and the resulting cDNA was fragmented with a sonicator (Covaris) using the following parameters: duty cycle 10%, peak power 175.0, cycles/burst 100, time 5 min.

Libraries were prepared for sequencing as previously described [35]. DNA was end repaired using T4 polymerase, Klenow fragment, and T4 polynucleotide kinase. 3′ A-tailing was performed using Exo-Klenow. NEBNext Illumina Multiplex Oligo Adaptors (NEB E7335S) were ligated for 1 h at room temperature. Unligated adapters were separated by gel electrophoresis (2.5% agarose, 0.5X TBE) and ligated DNA was purified using a NucleoSpin Gel Clean-up Kit (Macherey-Nagel). Ligated DNA was PCR amplified using NEBNext Multiplex Primers and purified using AMPure XP beads (Beckman Coulter). Purified libraries were quantified using Agilent 2100 Bioanalyzer HS DNA and multiplexed in equimolar concentrations. Sequencing was performed using an Illumina NextSeq at 1 × 75 bp by Stanford Functional Genomics Facility. RNA sequencing data is available through GEO (GSE115397).

Gene expression of patient-derived DIPG cultures and primary bulk DIPG tissue was published previously [11, 30, 35]. Single-cell RNA sequencing of diagnostic DIPG biopsies was also published previously [7]. All additional analysis was performed in R; FPKM plots were plotted in Prism (GraphPad).

### Gene expression analysis

Reads were mapped to hg19 annotation using Tophat2 [24]. Transcript coverage of RefSeq gene annotations were performed using featureCounts [25]. To visualize sample similarity between samples, we performed a regularized log-transformation using the R function rlog from DESeq2 [28], and plotted the PCA using ggplot2. Differential testing and log2 fold change calculation was performed using DESeq2, with FDR = 0.1 and normalization calculated by number of assigned reads. Active transcripts were defined as genes with a mean FPKM of at least 5 across all samples. Volcano plot was made in R. Gene Ontology on differentially expressed gene lists was performed using the DAVID v6.8 web portal [18, 19]. Pre-ranked gene set enrichment analysis was performed with the publically available software platform [33, 49].

### Human tissue culture

Authenticity of all cultures was routinely monitored and validated using short tandem repeat (STR) DNA fingerprinting, and tested regularly for mycoplasma. DIPG and adult GBM cultures were cultured as described previously [14, 35, 40]; briefly, all tumor cultures were maintained as neurospheres in Tumor Stem Media (TSM) consisting of DMEM/F12 (Invitrogen), Neurobasal(−A) (Invitrogen), B27(−A) (Invitrogen), human-bFGF (20 ng/ml) (Shenandoah Biotech, Warwick, PA), human-EGF (20 ng/ml) (Shenandoah), human PDGF-AB (20 ng/ml) (Shenandoah) and heparin (10 ng/ml) (STEMCELL Technologies). Human neural precursor cell cultures were maintained as neurospheres in DMEM/F12, Neurobasal(−A), B27(−A), human-bFGF, human-EGF, heparin, and LIF (Millipore).

### Luminex assay

Neurospheres were dissociated into a single cell suspension, and resuspended at 200 k cells/mL in 2.5 mL TSM without growth factors. After 24 h, supernatants were collected, centrifuged and filtered through a 0.22μm filter to remove cells, aliquoted and immediately stored at − 80 °C. All cultures were collected in triplicate at different cell passages. Supernatants were analyzed on the Luminex 200 platform by the Human Immune Monitoring Center at Stanford University. Human 63-plex kits were purchased from eBiosciences/Affymetrix and used according to the manufacturer’s recommendations with modifications. Briefly: Beads were added to a 96-well plate and washed in a Biotek ELx405 washer. Samples were added to the plate containing the mixed antibody-linked beads and incubated at room temperature for 1 h followed by overnight incubation at 4 °C with shaking. Cold and Room temperature incubation steps were performed on an orbital shaker at 500–600 rpm. Following the overnight incubation plates were washed in a Biotek ELx405 washer and then biotinylated detection antibody added for 75 min at room temperature with shaking. Plate was washed as above and streptavidin-PE was added. After incubation for 30 min at room temperature wash was performed as above and reading buffer was added to the wells. Each sample was measured in duplicate. Plates were read using a Luminex 200 instrument with a lower bound of 50 beads per sample per cytokine. Custom assay Control beads by Radix Biosolutions are added to all wells.

### Immunohistochemistry and light microscopy

Primary tumor samples were processed as previously described [34]. Briefly, primary tumor samples were fixed in 4% paraformaldehyde-PBS overnight, and then transferred to 30% sucrose until tissue samples sank (2–3 d). Tissue samples were transferred to cryomolds and embedded in optimal-cutting temperature (OCT) compound (TissueTek). 10 μm cryosections were generated on a cryostat (Leica). Endogenous peroxidase activity was neutralized (Bloxall, Vector Laboratories) before samples were permeabilized (0.5% Triton X-100, TBS) and blocked (5% horse serum, Vector). Immunohistochemical labeling was performed for CD3 (Biolegend, 300302, 3 h RT). CD3 was developed with a peroxidase secondary (ImmPRESS VR anti-rabbit IgG, Vector) and ImmPACT AMEC Red Peroxidase Substrate (Vector). Tissue samples were counterstained with Hematoxylin QS (Vector, 45 s, RT), developed in a bluing solution (0.1% NaCO_3_/H_2_O, 1 min) and coverslipped with Fluoro-Gel with Tris buffer (Electron Microscopy Services). Samples were imaged on a Nikon Eclipse.

### Immunofluorescence and confocal microscopy

Primary tumor samples were prepared as described for immunohistochemistry. Frozen cryosections were rehydrated in PBS. Antigen retrieval was performed with L.A.B. Solution (Polysciences, 5 min, RT). Sections were blocked for 1 h at RT in 3% NDS (Jackson ImmunoResearch) and stained overnight at 4 °C with rabbit anti-IBA1 (Wako, 1:1000). Secondary antibodies conjugated with AlexaFluor 488 were used at RT for 4 h to detect primary labeling (Jackson ImmunoResearch, 1:500). Sections were mounted in ProLong Gold Antifade Mountant with DAPI (Thermo Fisher). Mounted samples were images using confocal microscopy (Zeiss LSM710), and acquired Z stacks were flattened through maximum intensity projection (Zeiss ZEN).

## Results

**Fig. 1:**
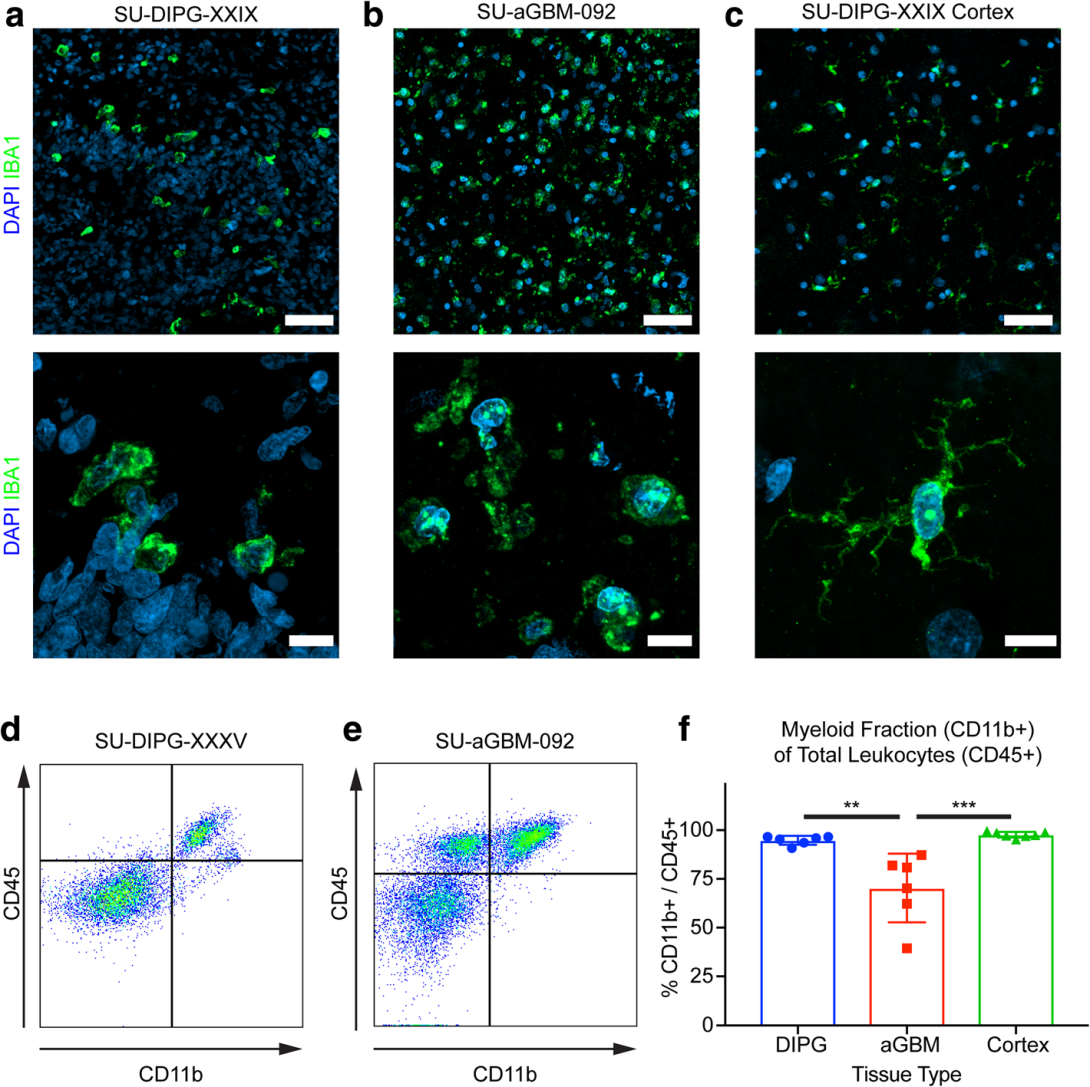
DIPG and aGBM tumor-associated microglia/macrophages (**a**-**c**) Immunofluorescence of primary DIPG tumor tissue (**a**), primary adult GBM tumor tissue (**b**), and primary pediatric cerebral cortex (**c**) for the myeloid cell marker IBA1 (green) and DAPI counterstain (blue). The tumor-associated macrophages in a-b exhibit an amoeboid morphology with shorter processes and enlarged cell bodies, while the normal cortical microglia in c demonstrates the ramified morphology typical of "resting" microglia. Top scale bar = 50μm; bottom scale bar = 10μm (**d**-**e**) Representative FACS plots showing CD11b expression against CD45 expression of primary DIPG (**d**) and aGBM (**e**) tissue. Samples were gated on size, singularity, and viability prior to these plots (**f**) Quantification of myeloid fraction (CD11b+) of total leukocytes (CD45+) as calculated by flow cytometry in DIPG, aGBM, and normal cortex primary tissue. ** *p* < 0.005, *** *p* < 0.0005 by t-test with Tukey multiple comparison correction

**Fig. 2:**
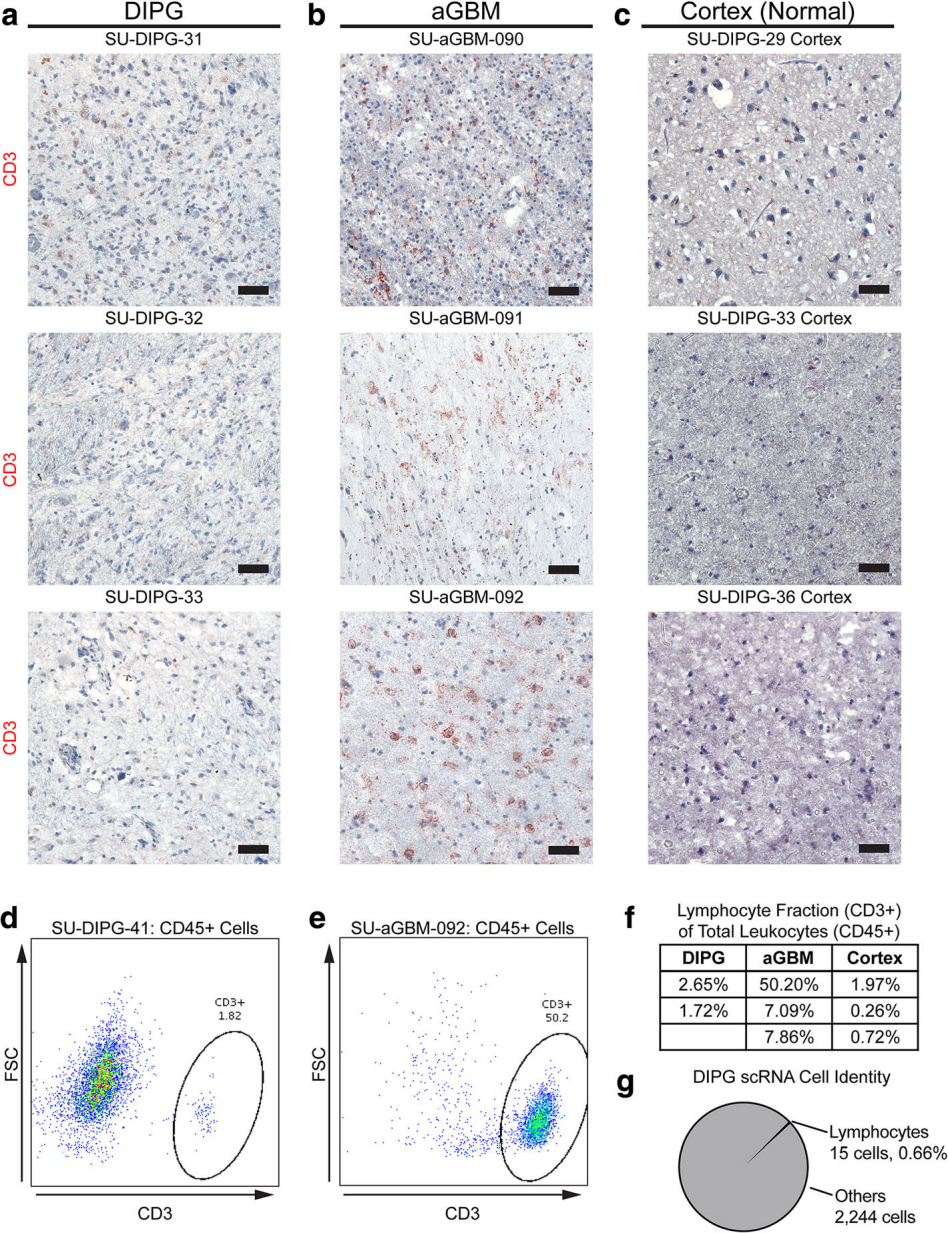
DIPG and aGBM demonstrate differential CD3+ lymphocyte infiltration (**a**-**c**) Immunohistochemical staining for CD3 (red) of primary DIPG (**a**), adult GBM (**b**), and pediatric cerebral cortex (**c**) tissue, counterstained with hematoxylin (blue). Scale bar = 50 μm (**d**-**e**) FACS plots showing different CD3+ composition of CD45+ cells in DIPG (**d**) and aGBM (**e**). Samples were gated for size, singularity, viability, and CD45 positivity prior to these plots (**f**) Table of lymphocyte (CD3+) fraction of total leukocytes (CD45+) in primary DIPG, aGBM, and pediatric cortical tissue samples as calculated via flow cytometry of primary dissociated early autopsy samples. **g** Lymphocyte fraction of all sampled cells from primary single-cell RNA sequencing of DIPG diagnostic biopsy samples [7]

We obtained primary DIPG tissue and pediatric normal cortical tissue samples at the time of early post-mortem autopsy (Additional file 1: Table S1). For single cell studies of DIPG, samples were obtained at the time of new diagnosis biopsy as previously described [8]. Adult GBM tissue samples were obtained from early post-mortem autopsy and from surgical biopsies. All tissue collection was performed with informed consent and IRB approval. Immunofluorescence staining of primary DIPG and adult GBM tissue demonstrates a marked increase of Iba1+ stained microglia/macrophages, which exhibit an activated morphology (shorter processes, enlarged cell bodies) when compared to microglia in normal cerebral cortex (Fig. 1a-c). To gain a better understanding of these DIPG-associated microglia/macrophages, we established a fluorescence-activated cell sorting (FACS) protocol for the isolation of CD11b+/CD45+ cells from early post-mortem autopsy tissue (Fig. 1d-e). Notably, while microglia and peripheral macrophages are sometimes differentiated by CD45 expression levels [1], we did not observe consistently variable CD45-positivity in primary DIPG samples (Additional file 2: Figure S1). We did observe a larger CD45+/CD11b- population in our adult GBM samples, indicating the presence of non-myeloid immune cells in adult GBM. Quantifying the percentage of CD45+ leukocytes that were CD11b + myeloid cells, we found a significant difference between DIPG and adult GBM samples (DIPG: 94.80% ± 0.92% vs. adult GBM: 70.34% ± 7.20%, *p* < 0.005, Fig. 1f). Nearly all CD45+ cells in normal pediatric cortical tissue were CD11b + myeloid cells (97.71% ± 1.48%), likely representing normal cortical microglia. As earlier reports exploring the immune compartments of other pediatric gliomas suggest that pediatric high-grade gliomas have limited lymphocytic infiltrate [15, 37], we performed immunohistochemical staining on our tissue samples for CD3 and found minimal CD3 staining in primary DIPG tissue compared to adult GBM, which may account for some of the CD45+/CD11b- population (Fig. 2a-c). Concordantly, flow cytometry revealed a small number of CD3+ lymphocytes in a representative cohort of DIPG samples compared to adult GBM samples (DIPG: 1.72–2.65% of total CD45+ leukocytes; adult GBM: 7.09–50.2%; normal cortex: 0.26–1.97%; Fig. 2d-f). In a separate set of samples, we examined unbiased single-cell RNA sequencing of H3K27M mutant pediatric diffuse midline glioma pre-treatment, diagnostic biopsy samples [8], and identified only 15 lymphocytes out of 2259 total live cells sequenced (0.66%) (Fig. 2g). These findings together provide further evidence for a minimal lymphocytic infiltrate in DIPG.

**Fig. 3:**
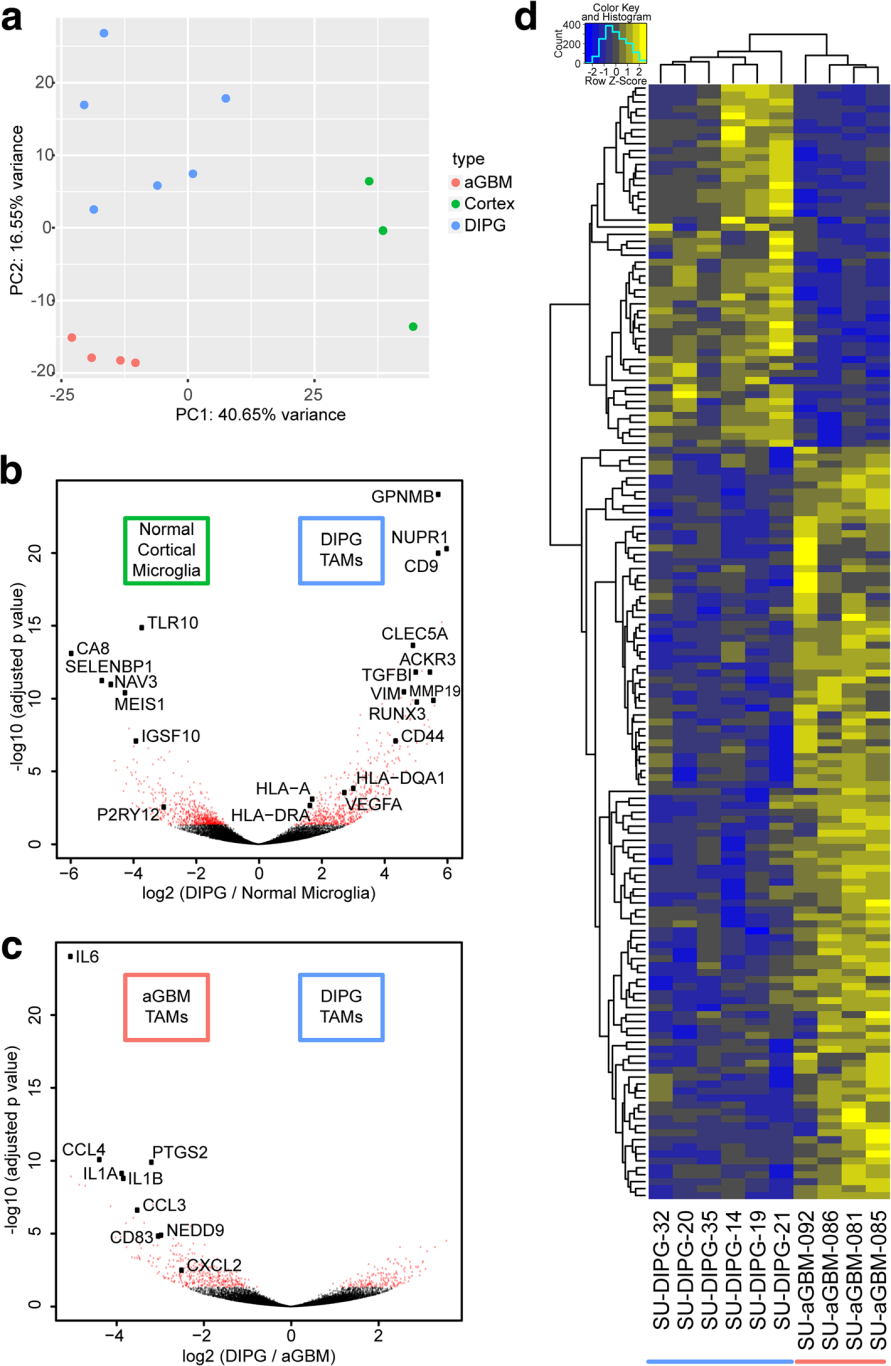
DIPG- and aGBM-associated macrophages exhibit distinct gene expression profiles (**a**) Principal components analysis of the top 500 varying genes across all samples demonstrates clusters corresponding to normal cortical microglia (green), DIPG-associated macrophages (blue), and aGBM-associated macrophages (red). **b**-**c** Volcano plot of log-fold change against adjusted *p* value for genes between normal cortical microglia and DIPG-associated macrophages (**b**), and between DIPG-associated macrophages and aGBM-associated macrophages (**c**). Red dots represent adjusted p value < 0.05, and selected significantly differentially expressed genes are identified (**d**) Heat map of normalized count values for differentially expressed genes between DIPG- and aGBM-associated macrophages. Hierarchical clustering demonstrates a distinct difference between the two groups

To further examine and compare the DIPG- and aGBM-associated macrophage phenotype, we performed gene expression analyses. First, we isolated CD11b+/CD45+ cells from broadly sampled primary DIPG autopsy samples and aGBM tumor autopsy or surgical biospy samples by FACS, verified population purity by flow cytometry, and lysed cells in Trizol for RNA sequencing. After filtering samples for RNA quality, we included six DIPG autopsy, four adult GBM (three surgical biopsy and one autopsy sample), and three pediatric autopsy cortical control samples in our final analyses. These samples all exhibited robust enrichment for microglia/macrophage-specific genes, validating the FACS-based myeloid cell isolation strategy (Additional file 3: Figure S2). Principal components analysis of the top 500 varying genes amongst these samples demonstrated three distinct populations, with the first principal component primarily separating normal cortex myeloid cells from both adult GBM and DIPG myeloid cell samples, and the second principal component primarily separating DIPG and adult GBM myeloid cell samples (Fig. 3a). Gene ontology (GO) analysis of the top 50 genes enriched in DIPG and adult GBM myeloid cell samples in PC1 revealed biological process terms including *cell adhesion, angiogenesis,* and *extracellular matrix organization* (Table 1). GO analysis of the top 50 genes enriched in adult GBM myeloid cell samples in PC2 identified terms including *monocyte chemotaxis, inflammatory response, immune response, neutrophil chemotaxis, cellular response to interleukin-1,* and *chemokine-mediated signaling pathway*, which may suggest that DIPG- and adult GBM-associated macrophages are differentially inflammatory (Table 1).

**Table 1:** Gene-ontology terms associated with the top 50 genes contributing to principal component 1 (top) or principal component 2 (bottom)

GO Terms: Top genes down in PC1 (up in DIPG/aGBM)		
GO Term	*p*-value	Genes
Cell adhesion	3.178E-08	*IBSP, CD9, PLXNC1, CD44, TGFBI, VCAN, ACKR3, THBS1, GPNMB, EMILIN2, CD72, FN1*
Angiogenesis	2.285E-06	*VAV3, EREG, TGFBI, MMP19, CXCL8, ACKR3, ANPEP, FN1*
Extracellular matrix organization	1.488E-05	*IBSP, CD44, DMP1, TGFBI, VCAN, THBS1, FN1*
Platelet degranulation	1.787E-04	*CD9, ACTN1, THBS1, FLNA, FN1*
Peptide cross-linking	3.446E-04	*ANXA1, TGM2, THBS1, FN1*
Neutrophil chemotaxis	7.802E-04	*VAV3, LGALS3, CXCL8, TREM1*
Movement of cell or subcellular component	1.679E-03	*CD9, VIM, CXCL8, FPR3*
Platelet activation	3.834E-03	*CD9, VAV3, ACTN1, FLNA*
GO Terms: Top genes down in PC2 (up in aGBM)		
GO Term	p-value	Genes
Monocyte chemotaxis	3.14E-08	*IL6, CCL3, CCL2, CCL3L1, CCL4L2, CCL4*
Inflammatory response	9.84E-08	*PLP1, IL6, CCL3, CCL2, CCL3L1, CXCL9, IL1B, CCL4L2, CCL4, IL1A*
Immune response	2.40E-07	*IL6, CCL3, CCL2, ENPP2, FCGR1B, CXCL9, IL1B, CCL4L2, CCL4, IL1A*
Neutrophil chemotaxis	3.18E-07	*CCL3, CCL2, CCL3L1, IL1B, CCL4L2, CCL4*
Cellular response to interleukin-1	4.60E-07	*IL6, CCL3, CCL2, CCL3L1, CCL4L2, CCL4*
Chemokine-mediated signaling pathway	4.60E-07	*CCL3, CCL2, CCL3L1, CXCL9, CCL4L2, CCL4*
Positive regulation of ERK1 and ERK2 cascade	2.09E-06	*IL6, CCL3, CCL2, CCL3L1, CCL4L2, FGF1, CCL4*
Cellular response to tumor necrosis factor	4.06E-06	*IL6, CCL3, CCL2, CCL3L1, CCL4L2, CCL4*

We next performed differential gene testing between our sample groups using DESeq2 (Fig. 3b-c). After filtering for significance (adjusted *p*-value < 0.1) and minimum gene expression (mean FPKM > 5 across all samples), there were 330 differentially expressed genes between DIPG and control (176 up, 154 down); 203 between aGBM and control (164 up, 39 down); and 160 between aGBM and DIPG (108 up, 52 down) (Additional file 4: Table S2). Macrophage response phenotypes have historically been separated into M1 “classical activation” and M2 “alternative activation” states, in which M1 macrophages may represent an anti-tumor response whereas M2 macrophages provide pro-tumorigenic functions, although this delineation is widely held to be insufficient to capture the diversity of macrophage phenotypes [31]. Previous reports have suggested that adult GAMs span the spectrum of M1 and M2 phenotypes [10]. We performed pre-ranked gene-set enrichment analysis on significant differentially expressed genes between DIPG-associated macrophages and cortical microglia and similarly found no significant enrichment for either M1 or M2 defined gene sets [32] (Additional file 5: Figure S3A-B), indicating that DIPG-associated macrophages do not fit neatly into an “M1” or “M2” classification.

We also examined the differentially expressed gene lists by GO analysis. GO analysis of genes upregulated in DIPG-associated macrophages compared to cortical microglia include the terms *interferon-gamma-mediated signaling pathway*, *response to hypoxia*, *antigen processing and presentation* (e.g. HLA-A, HLA-DQA1, HLA-DRA), *signal transduction*, *type I interferon signaling pathway,* and *positive regulation of angiogenesis*. GO analysis of genes downregulated in DIPG-associated macrophages compared to cortical microglia include *MyD88-dependent toll-like receptor signaling pathway*, *regulation of cytokine secretion*, and *toll-like receptor signaling pathway* (Additional file 5: Figure S3C-D). This suggests that DIPG-associated macrophages exhibit some degree of activation, consistent with the observed morphological changes (Fig. 1a-c). However, the top genes upregulated in adult GBM-associated macrophages compared to DIPG-associated macrophages include many inflammation-associated genes (e.g. IL6, CCL4, IL1A, IL1B, CCL3, PTGS2) (Fig. 3c). GO analysis of genes upregulated in adult GBM-associated macrophages compared to DIPG-associated macrophages included the terms *inflammatory response*, *positive regulation of smooth muscle cell proliferation*, *cellular response to interleukin-1*, *positive regulation of transcription from RNA polymerase II promoter*, *immune response*, *cellular response to tumor necrosis factor*, and *chemokine-mediated signaling pathway* (Table 2). Overall, the gene expression profile of adult GBM-associated macrophages and DIPG-associated macrophages are markedly different (Fig. 3d).

**Table 2:** Gene-ontology terms upregulated in adult GBM tumor-associated macrophages compared to DIPG tumor-associated macrophages

GO Terms upregulated in aGBM TAMs vs. DIPG TAMs		
GO Term	*p*-value	Genes
Inflammatory response	4.70E-13	NFKBIZ, IL6, CCL3, OLR1, PTGS2, CXCL2, CXCL8, NFKB1, NLRP3, CCL5, CCL4, CCRL2, FOS, IL1B, NAIP, CLEC7A, NFE2L2, TNFAIP3, IL1A
Positive regulation of smooth muscle cell proliferation	5.66E-07	NAMPT, IL6, EREG, PTGS2, HBEGF, NR4A3, CCL5
Cellular response to interleukin-1	1.55E-06	ICAM1, IL6, CCL3, CXCL8, NFKB1, CCL5, CCL4
Positive regulation of transcription from RNA polymerase II promoter	1.57E-06	EGR1, NAMPT, IL6, EGR2, CCNH, NR4A2, CCNL1, NFKBIA, NFKB1, FOSB, NR4A3, NLRP3, FOS, ATF3, RGCC, IL1B, NFE2L2, IL1A, KLF4
Immune response	8.70E-06	GPR183, IL6, CCL3, CXCL2, IL1RN, CXCL8, IL1B, CCL5, SLED1, CCL4, GBP2, IL1A
Cellular response to tumor necrosis factor	1.98E-05	ICAM1, IL6, CCL3, CXCL8, NFE2L2, CCL5, CCL4
Chemokine-mediated signaling pathway	2.96E-05	CCRL2, CCL3, CXCL2, CXCL8, CCL5, CCL4
Positive regulation of gene expression	5.38E-05	IL6, LIMS1, CCL3, RGCC, MDM2, IL1B, NFE2L2, KLF4, IL1A

**Fig. 4:**
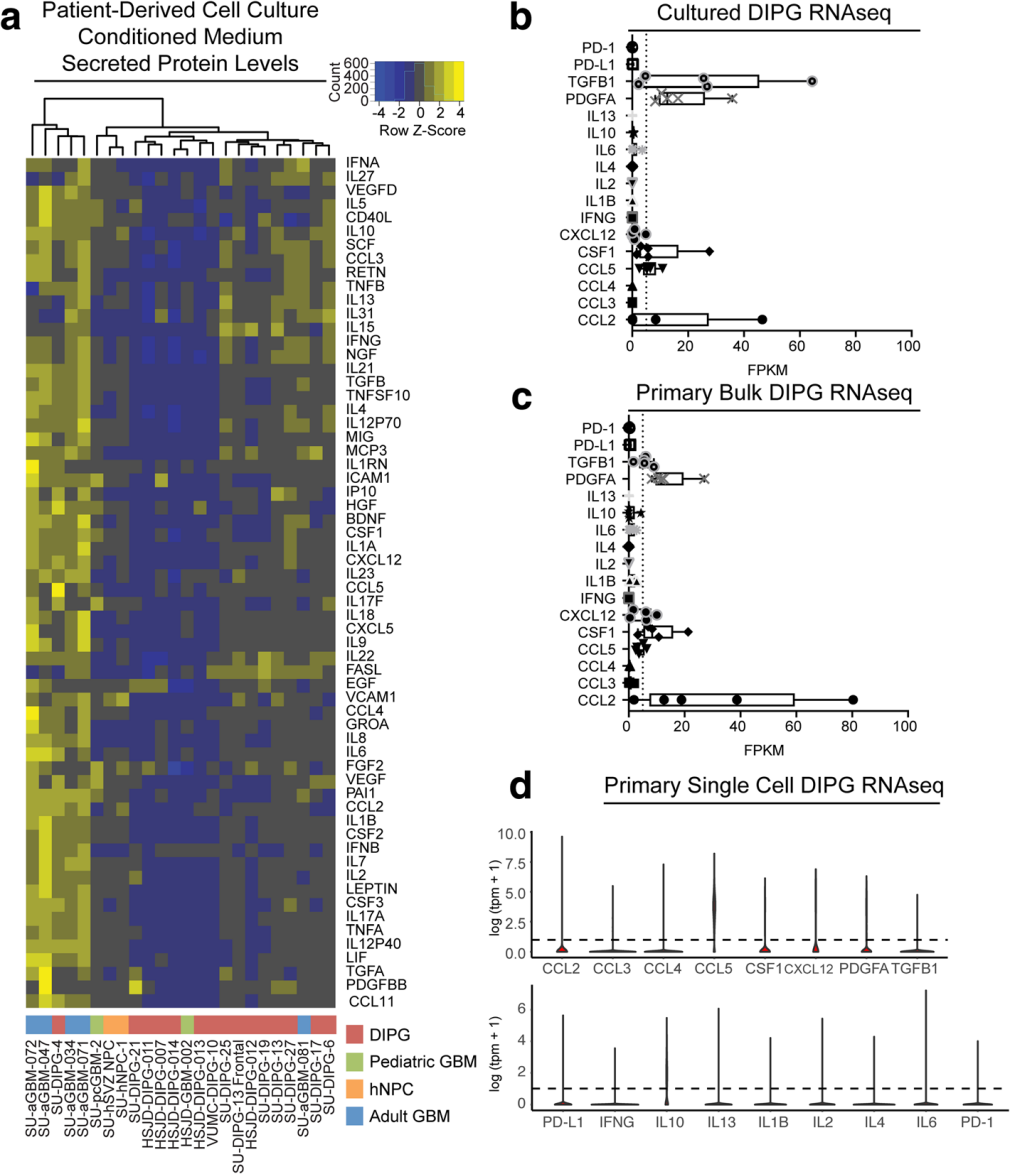
Patient-derived DIPG and aGBM cell cultures exhibit distinct cytokine secretion profiles (**a**) Hierarchical clustering of mean fluorescence intensity values of secreted factors in conditioned medium derived from patient-derived DIPG (red), aGBM (blue), pediatric GBM (green), and human neural precursor cell (hNPC) cultures (orange). Each column represents the average of samples tested in triplicate, and each row represents a separate measured cytokine (**b**-**c**) Box and whisker plot of FPKMs of cytokines and chemokines from patient-derived DIPG cell cultures (**b**) and primary bulk DIPG tissue (**c**) RNA sequencing data [11, 30, 35]. Dashed line represents FPKM = 5 (**d**) Violin plots of gene expression from single-cell RNA sequencing of diagnostic DIPG biopsy samples [7]. Dashed line represents log(tpm + 1) = 1

Glioma-secreted factors play a role in modulating GAMs and the glioma immune microenvironment [12]. Given that our data suggest DIPG-associated macrophages are less inflammatory than adult GBM-associated macrophages, we asked whether DIPG-derived factors might differ from adult GBM-derived factors. We collected conditioned medium from 14 patient-derived DIPG cultures, a DIPG culture derived from a frontal lobe metastasis (SU-DIPG-XIII-Frontal), two patient-derived pediatric GBM cultures, two human neural precursor cell cultures, and five patient-derived adult GBM cultures and analyzed their cytokine/chemokine secretome using an ELISA array (Luminex 200). Hierarchical clustering analysis showed that the majority of DIPG and pediatric GBM cultures were more like human neural precursor cells and secrete substantially fewer cytokines and chemokines than adult GBM cells (Fig. 4a). Given the low level of DIPG-derived factors, we looked for gene expression of these factors in our previously published patient-derived DIPG cell culture RNA sequencing data [35, 40] and found that patient-derived DIPG cell cultures do not express cytokine genes, and only express a limited number of chemokines and growth factors (Fig. 4b, Additional file 6: Figure S4A). Exploring the same genes in primary DIPG tissue bulk RNA sequencing data [14] similarly demonstrated low levels of these cytokines in the overall DIPG tumor microenvironment (Fig. 4c, Additional file 6: Figure S4B). Finally, we explored these genes in a recent single-cell gene expression dataset that examined primary diagnostic biopsy tissue from DIPG and other H3K27M-mutant diffuse midline gliomas [8], which enables investigating individual DIPG cells in the primary, pre-treatment tumor microenvironment (Fig. 4d, Additional file 6: Figure S4C). DIPG tumor cell gene expression data from each of these datasets support the observation that DIPG cells only express a small subset of chemokines and growth factors. Gene transcripts or proteins found in at least three of these four analyses included CCL2, CCL5, CSF1, CXCL12, TGFB1, and PDGFA. Notably, we find that DIPG cells do not express inflammatory cytokines and chemokines that may recruit other immune cells to the tumor microenvironment. For instance, the lack of IL2 expression by DIPG cells may contribute the lack of T-lymphocyte infiltration observed. Moreover, examination of these gene expression datasets for the immunomodulatory ligands PD-1 and PD-L1 indicated no expression (Fig. 4b-d, Additional file 6: Figure S4).

## Discussion

The data presented here indicate that DIPG-associated macrophages are strikingly less inflammatory compared to adult GBM-associated macrophages, expressing markedly lower levels of IL6, IL1A, IL1B, CCL3, CCL4, among other inflammatory factors. Concordantly, we find that DIPG tumor cells in culture, primary autopsy tissue, and pre-treatment diagnostic DIPG biopsy samples, are also non-inflammatory and lack expression of most chemokines and cytokines. In fact, one of the few factors expressed by DIPG is TGFB1, a known immunosuppressive growth factor. We also observe a lack of infiltrating CD3+ lymphocytes in primary DIPG tissue, suggesting that the presence of DIPG does not recruit tumor-infiltrating lymphocytes (TILs). Importantly, we observed this both in early post-mortem DIPG autopsy samples as well as pre-treatment diagnostic DIPG biopsy samples, suggesting that TILs are not a meaningful part of early or late DIPG pathophysiology.

Historically, macrophages have been classified as “M1” classically activated or “M2” alternatively activated phenotypes, although this classification system has been acknowledged to be insufficient to capture the complexity of macrophage responses [31]. Although DIPG tumor cells produce CSF1, a cytokine associated with the M2, pro-tumorigenic phenotype, we found that DIPG-associated macrophages do not fit neatly into an M1 or M2 classification [32]. Similar to reports in adult GBM [10], DIPG-associated macrophages appear to be in a tumor-specific activation phenotype related to the distinct tumor-derived chemokine milieu. In our analysis of DIPG-associated macrophage gene expression, we observed that these cells express increased levels of antigen-presentation genes such as HLA proteins. However, the comparative lack of production of pro-inflammatory chemokines (e.g. CCL3, CCL4) and absence of lymphocytes in primary tissue are consistent with the failure of DIPG-associated macrophages to trigger an effective anti-tumor immune response. Adding to the evidence for lack of an effective innate or adaptive immune response in pediatric high-grade gliomas, a previous study demonstrated the lack of NK cell infiltration into pediatric high-grade gliomas [16], although this study did not specifically investigate DIPG. An “immune cold” state of DIPG is also consistent with the lack of inflammatory cells in pediatric non-brainstem gliomas recently described [30].

The findings presented here are particularly relevant to the development of immunotherapeutic approaches to DIPG. Many current approaches in adult GBM include the use of checkpoint inhibitors [36], the effectiveness of which is linked to pre-existing CD8+ T cell presence [51] and mutational load [42]. Along with our observation that DIPG tumors contain very few infiltrating T-cells, DIPG exhibits a lower mutational burden compared to adult glioblastoma [47]. Thus, immunotherapy approaches in DIPG may be better served by focusing on inducing recruitment or introduction of immune cells to the tumor. One promising strategy involves the use of chimeric antigen receptor T (CAR-T) cells, which are designed to recognize tumor-associated antigens. We recently demonstrated striking preclinical efficacy of GD2-targeted CAR-T cell therapy in preclinical models of DIPG [34].

## Conclusion

Adult and pediatric high-grade gliomas are distinct disease entities, and differences between DIPG and adult glioblastoma extend to the immunological phenotype of the tumor microenvironment. In contrast to adult GBM, the immune microenvironment of DIPG is non-inflammatory and does not contain a significant adaptive immune component. These observations provide important considerations for the design of immunotherapeutic approaches for DIPG.

## Additional files

Additional file 1: **Table S1.** Patient characteristics of early post-mortem DIPG autopsy cases. (XLSX 55 kb)

Additional file 2: **Figure S1.** Primary DIPG samples do not consistently demonstrate differential CD45 high/low populations (a-b) Representative FACS plots of primary DIPG tissue samples showing an example of an indistinguishable CD45 high/low sample (a) and a distinguishable CD45 high/low population (b). Samples were gated for size, singularity, and viability prior to these plots. (TIF 585 kb)

Additional file 3: **Figure S2.** Isolated microglia/macrophages are enriched for myeloid genes. FPKMs binned across sample type for isolated DIPG (blue), aGBM (red), and pediatric cortical microglia/macrophages (green). There is minimal or absent expression of genes associated with other major cortical cell types (astrocytes, neurons, OPCs, oligodendrocytes, and endothelial cells). (TIF 948 kb)

Additional file 4: **Table S2.** Significant differentially expressed genes between normal cortical microglia, DIPG-associated macrophages, and aGBM-associated macrophages. (XLSX 75 kb)

Additional file 5: **Figure S3.** DIPG-associated macrophages are not M1 or M2 (a-b) Pre-ranked gene set enrichment analysis of significantly differentially regulated genes between DIPG-associated macrophages and normal cerebral cortex microglia compared against published gene sets corresponding to M1 (a) or M2 (b) macrophage polarization state [27] (c-d) GO term analysis of upregulated (c) and downregulated (d) genes in DIPG-associated macrophages compared to cortical microglia. (TIF 2553 kb)

Additional file 6: **Figure S4.** DIPG cells do not express significant levels of cytokines (a-b) FPKMs of cytokine (left), chemokine (middle) and other factors (right) expressed by patient-derived DIPG cell cultures (a) or in bulk primary DIPG tissue (b) Horizontal line represents FPKM = 5 (c) Violin plots of single-cell DIPG expression of cytokines, chemokines, and other factors from primary DIPG biopsy tissue. Horizontal line represents log(tpm + 1) = 1. (TIF 1442 kb)
