# Supplementary figures and images for: Non-inflammatory tumor microenvironment of diffuse intrinsic pontine glioma
Source: Acta Neuropathol Commun. 2018 Jun 28;6:51. doi: 10.1186/s40478-018-0553-x (PMC6022714; doi:10.1186/s40478-018-0553-x)

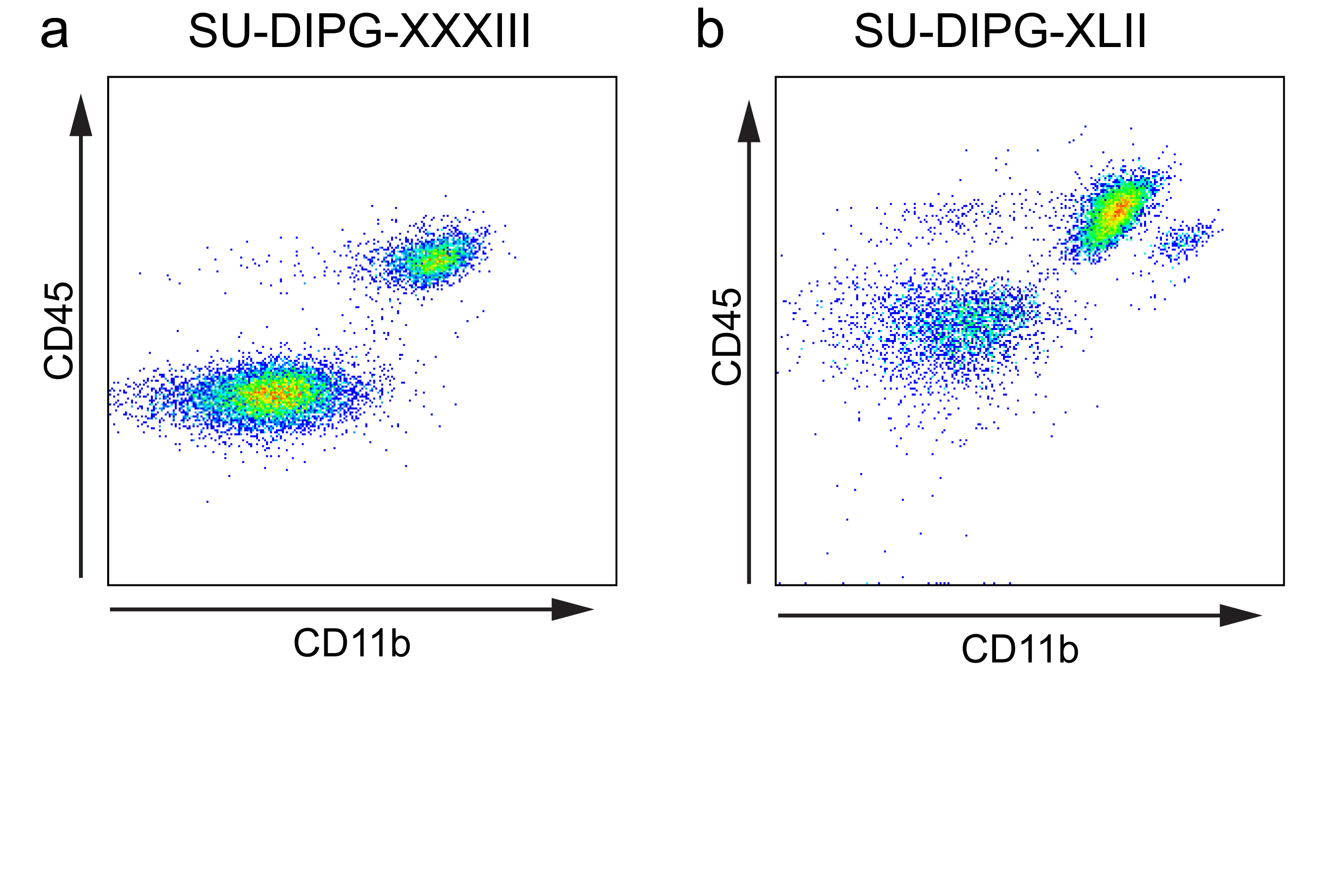

Supplement: Supplementary file 2 — Figure S1. Primary DIPG samples do not consistently demonstrate differential CD45 high/low populations (a-b) Representative FACS plots of primary DIPG tissue samples showing an example of an indistinguishable CD45 high/low sample (a) and a distinguishable CD45 high/low population (b). Samples were gated for size, singularity, and viability prior to these plots. (TIF 585 kb) [file 40478_2018_553_MOESM2_ESM.tif]

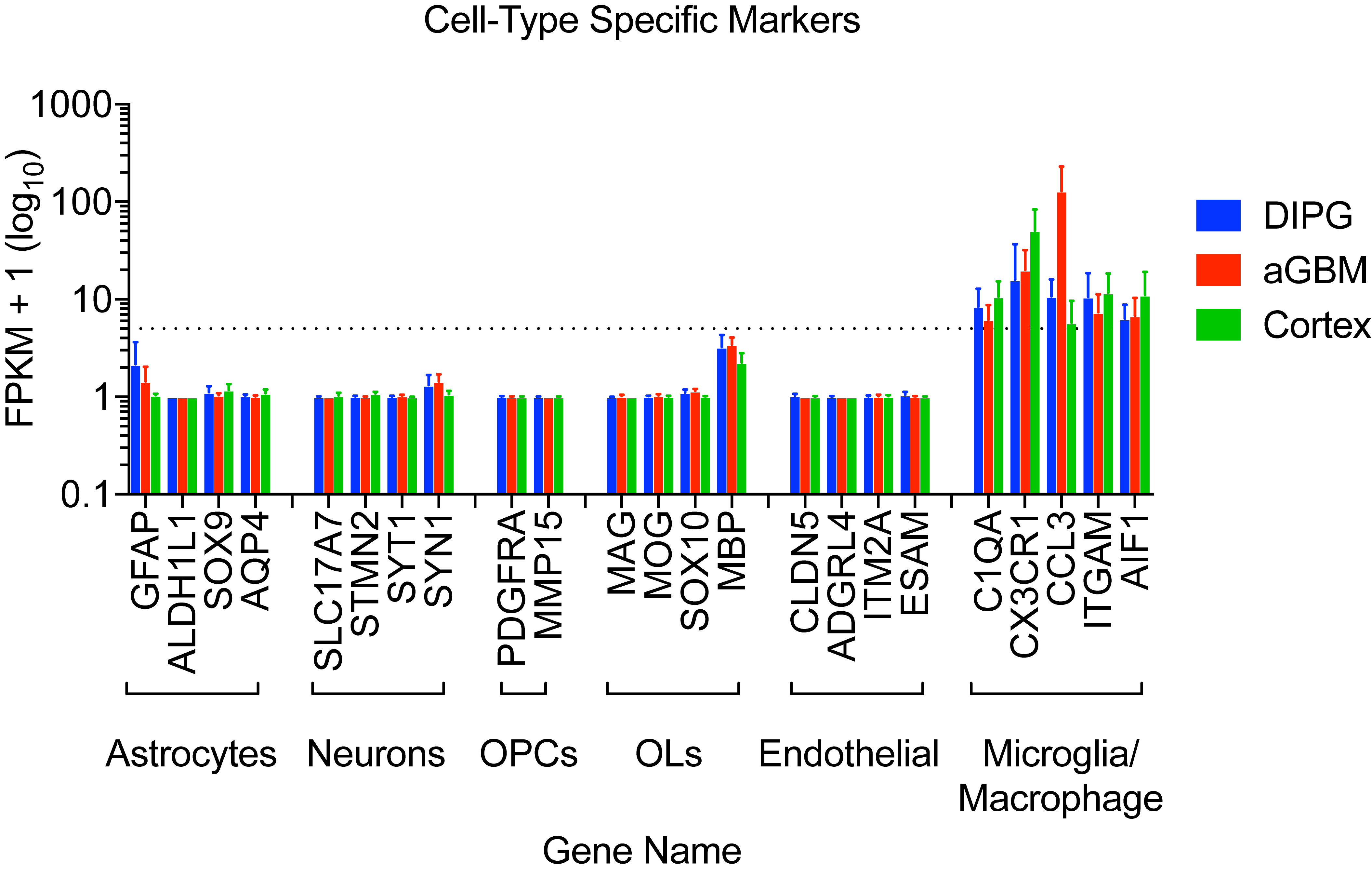

Supplement: Supplementary file 3 — Figure S2. Isolated microglia/macrophages are enriched for myeloid genes. FPKMs binned across sample type for isolated DIPG (blue), aGBM (red), and pediatric cortical microglia/macrophages (green). There is minimal or absent expression of genes associated with other major cortical cell types (astrocytes, neurons, OPCs, oligodendrocytes, and endothelial cells). (TIF 948 kb) [file 40478_2018_553_MOESM3_ESM.tif]

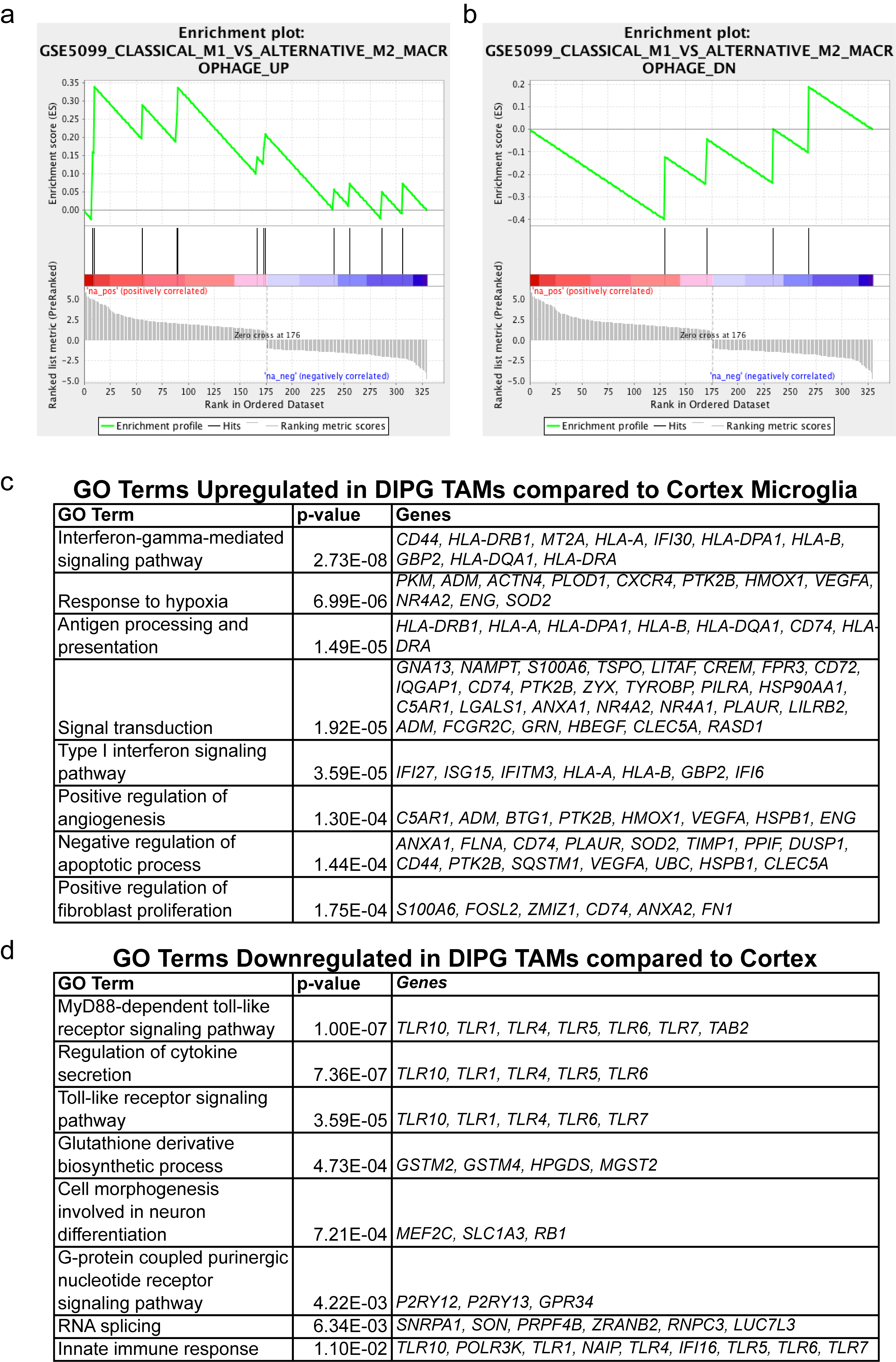

Supplement: Supplementary file 5 — Figure S3. DIPG-associated macrophages are not M1 or M2 (a-b) Pre-ranked gene set enrichment analysis of significantly differentially regulated genes between DIPG-associated macrophages and normal cerebral cortex microglia compared against published gene sets corresponding to M1 (a) or M2 (b) macrophage polarization state [27] (c-d) GO term analysis of upregulated (c) and downregulated (d) genes in DIPG-associated macrophages compared to cortical microglia. (TIF 2553 kb) [file 40478_2018_553_MOESM5_ESM.tif]

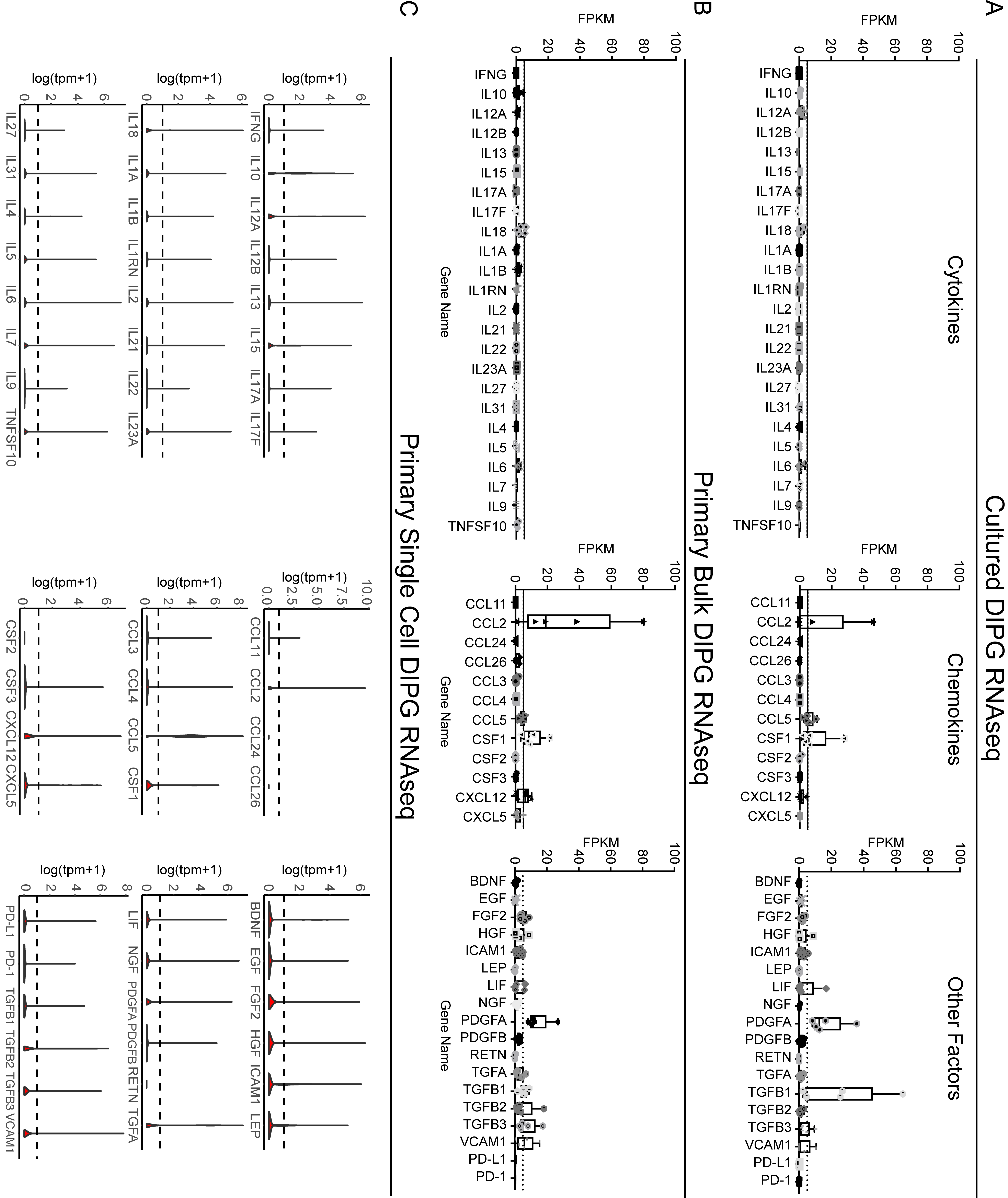

Supplement: Supplementary file 6 — Figure S4. DIPG cells do not express significant levels of cytokines (a-b) FPKMs of cytokine (left), chemokine (middle) and other factors (right) expressed by patient-derived DIPG cell cultures (a) or in bulk primary DIPG tissue (b) Horizontal line represents FPKM = 5 (c) Violin plots of single-cell DIPG expression of cytokines, chemokines, and other factors from primary DIPG biopsy tissue. Horizontal line represents log(tpm + 1) = 1. (TIF 1442 kb) [file 40478_2018_553_MOESM6_ESM.tif]
